# Council of State and Territorial Epidemiologists/CDC Surveillance Case Definition for Multisystem Inflammatory Syndrome in Children Associated with SARS-CoV-2 Infection — United States

**DOI:** 10.15585/mmwr.rr7104a1

**Published:** 2022-12-16

**Authors:** Michael Melgar, Ellen H. Lee, Allison D. Miller, Sarah Lim, Catherine M. Brown, Anna R. Yousaf, Laura D. Zambrano, Ermias D. Belay, Shana Godfred-Cato, Joseph Y. Abrams, Matthew E. Oster, Angela P. Campbell

**Affiliations:** ^1^COVID-19 Response Team, CDC, Atlanta, Georgia; ^2^General Surveillance Unit, Bureau of Communicable Disease, New York City Department of Health and Mental Hygiene, Queens, New York; ^3^Minnesota Department of Health, St. Paul, Minnesota; ^4^Massachusetts Department of Public Health, Boston, Massachusetts; ^5^School of Medicine, Emory University, Atlanta, Georgia; ^6^Children’s Healthcare of Atlanta, Atlanta, Georgia

## Abstract

**Since May 14, 2020, CDC has conducted national surveillance for multisystem inflammatory syndrome in children (MIS-C) associated with infection with SARS-CoV-2, the virus that causes COVID-19, among persons aged <21 years using a CDC case definition based on public health need and data from early reports of patients with this condition. Recent analyses of accumulated data indicated that certain criteria from the 2020 CDC MIS-C case definition performed better than others in distinguishing MIS-C from other illnesses and that certain other criteria likely contributed to misclassification. To incorporate lessons learned from MIS-C surveillance and public health investigations and to improve feasibility of implementation by surveillance staff at state, local, territorial, and tribal health departments, the Council of State and Territorial Epidemiologists (CSTE) and CDC developed a CSTE/CDC position statement (approved by CSTE in 2022) that includes an MIS-C surveillance case definition for voluntary reporting to CDC (effective January 1, 2023):**

**This report summarizes the evidence and rationale supporting the components of the CSTE/CDC MIS-C surveillance case definition and describes the methods used to develop the definition. These methods included convening MIS-C clinical experts (i.e., consultants):**

*regarding identification of MIS-C and its distinction from other pediatric conditions, a review of available literature comparing MIS-C phenotype with that of pediatric COVID-19 and other hyperinflammatory syndromes, and retrospective application of different criteria to data from MIS-C cases previously reported to CDC.*

**The CSTE/CDC surveillance case definition for MIS-C includes four important changes, in comparison with the 2020 CDC MIS-C case definition. These changes are 1) no required duration of subjective or measured fever; 2) requirement of C-reactive protein ≥3.0 mg/dL to indicate systemic inflammation; 3) adjustments to criteria of organ system involvement to include addition of shock as a separate category and elimination of respiratory, neurologic, and renal criteria; and 4) new requirements on timing of positive SARS-CoV-2 laboratory testing relative to the MIS-C illness. Although MIS-C is not a nationally notifiable condition and reporting is voluntary, CSTE and CDC recommend that all states and territories report all cases meeting confirmed, probable, or suspect criteria of the CSTE/CDC MIS-C surveillance case definition beginning January 1, 2023, for cases with MIS-C illness onset on or after that date:**

## Introduction

Multisystem inflammatory syndrome in children (MIS-C) is a severe hyperinflammatory condition occurring 2–6 weeks after infection with SARS-CoV-2, the virus that causes COVID-19 (*1*). On May 14, 2020, in response to the first identified MIS-C illnesses in Europe and the United States (*2*–*6*), CDC released a health advisory outlining an MIS-C case definition and soliciting reports from clinicians (*7*). On the basis of limited data from early investigations, this initial case definition was intentionally broad to capture as many potential cases as possible, and it has been used for approximately 2.5 years of national passive case-based surveillance of MIS-C (*8*–*11*). As of November 30, 2022, approximately 9,000 cases of illness in persons aged <21 years meeting the 2020 CDC MIS-C case definition had been reported in the United States through national MIS-C surveillance (*12*).

Because of urgent public health need early in the pandemic, the 2020 CDC MIS-C case definition was not reviewed or voted on by the Council of State and Territorial Epidemiologists (CSTE). Concurrently, the World Health Organization (WHO), the Brighton Collaboration, and the Royal College of Paediatrics and Child Health in the United Kingdom also developed MIS-C case definitions for surveillance or clinical guidance, based on early clinical data. These definitions differed with respect to patient age limits, fever duration, and organ system involvement (*13*–*15*) (Supplementary Table 1, https://stacks.cdc.gov/view/cdc/121710). Development of an MIS-C case definition for national surveillance is challenging because of a lack of a confirmatory diagnostic laboratory test or pathognomonic clinical features that distinguish MIS-C from COVID-19 or other pediatric hyperinflammatory syndromes such as Kawasaki disease. As a result, the 2020 CDC MIS-C case definition is complex, relying on combinations of clinical features and supporting laboratory tests to establish inclusion criteria. However, review of the 2020 CDC MISC case definition identified areas where criteria could be simplified by clarifying the minimum required fever duration, reducing open-ended lists of organ-specific clinical criteria to a limited list of the most important MIS-C features, and narrowing the laboratory tests used as evidence of systemic inflammation. An additional consideration was to reduce misclassification. Recent analyses, including from the Overcoming COVID-19 Network MIS-C registry, a CDC-funded pediatric hospital surveillance network registry of MIS-C patients, have indicated that certain criteria from the 2020 CDC MIS-C case definition performed better than others in distinguishing MIS-C from other conditions (*16*,*17*).

A joint CSTE/CDC work group* was established to develop a CSTE position statement for a standardized MIS-C surveillance case definition with reduced risk for misclassification and reduced complexity for better ease of implementation. The position statement was approved at the CSTE Annual Conference on June 23, 2022 (*18*). The purpose of this report is to describe the process and considerations that contributed to components of the CSTE/CDC MIS-C surveillance case definition and to encourage health departments to report all cases meeting confirmed, probable, or suspect criteria to CDC.

### CSTE/CDC Surveillance Case Definition for MIS-C

During the CSTE Annual Conference on June 23, 2022, CSTE voted and approved the position statement entitled “Standardized Case Definition for Surveillance of Multisystem Inflammatory Syndrome in Children Associated with SARS-CoV-2 Infection” that includes an MIS-C surveillance case definition (hereafter referred to as the CSTE/CDC MIS-C surveillance case definition) and criteria to identify an incident MIS-C case in a person aged <21 years (Box). A comparison of criteria in the CSTE/CDC MIS-C surveillance case definition and the 2020 CDC MIS-C case definition is available (Supplementary Table 2, https://stacks.cdc.gov/view/cdc/121710).

BOX **Council of State and Territorial Epidemiologists/CDC surveillance case definition for multisystem inflammatory syndrome (MIS-C) in children associated with SARS-CoV-2 infection — United States**

**Case definition classifications**

**Confirmed:** Meets the clinical criteria and the laboratory criteria.

**Probable:** Meets the clinical criteria and the epidemiologic linkage criteria.

**Suspect:** Meets the vital records criteria.

**Clinical criteria**

An illness in a person aged <21 years characterized by all of the following, in the absence of a more likely alternative diagnosis*:

- Subjective or documented fever (temperature ≥38°C)
- Clinical severity requiring hospitalization or resulting in death
- Evidence of systemic inflammation (indicated by C-reactive protein of ≥3.0 mg/dL [30 mg/L])
- New onset manifestations in at least two of the following categories:
  - Cardiac involvement (indicated by left ventricular ejection fraction of <55%; coronary artery dilatation, aneurysm, or ectasia; or troponin elevated above laboratory normal range, or indicated as elevated in a clinical note)
  - Mucocutaneous involvement (indicated by rash, inflammation of the oral mucosa [e.g., mucosal erythema or swelling, drying or fissuring of the lips, strawberry tongue], conjunctivitis or conjunctival injection [redness of the eyes], or extremity findings such as erythema [redness] or edema [swelling] of the hands or feet)
  - Shock^†^
  - Gastrointestinal involvement (indicated by abdominal pain, vomiting, or diarrhea)
  - Hematologic involvement (indicated by platelet count of <150,000 cells/*μ*L or absolute lymphocyte count of <1,000 cells/*μ*L)

**Laboratory criteria**

- Detection of SARS-CoV-2 RNA in a clinical specimen^§^ up to 60 days before or during hospitalization, or in a postmortem specimen using a diagnostic molecular amplification test (e.g., polymerase chain reaction); or
- Detection of SARS-CoV-2–specific antigen in a clinical specimen^§^ up to 60 days before or during hospitalization, or in a postmortem specimen; or
- Detection of SARS-CoV-2–specific antibodies^¶^ in serum, plasma, or whole blood associated with current illness resulting in or during hospitalization

**Epidemiologic linkage criteria**

Close contact** with a confirmed or probable case of COVID-19 disease in the 60 days before hospitalization.

**Vital records criteria**

A death of a person aged <21 years whose death certificate lists MIS-C or multisystem inflammatory syndrome as an underlying cause of death or a significant condition contributing to death.

**Criteria to distinguish a new case from an existing case**

A case should be enumerated as a new case if the person had never been enumerated as a case or if the person was most recently enumerated as a case with illness onset date (if available) or hospital admission date >90 days previous.

* If documented by the clinical treatment team, a final diagnosis of Kawasaki disease should be considered an alternative diagnosis. These cases should not be reported to national MIS-C surveillance.

^†^ Clinician documentation of shock meets this criterion.

^§^ Positive molecular or antigen results from self-administered testing using over-the-counter test kits meet laboratory criteria.

^¶^ Includes a positive serology test regardless of COVID-19 vaccination status. Detection of anti-nucleocapsid antibody is indicative of SARS-CoV-2 infection, whereas anti-spike protein antibody might be induced either by COVID-19 vaccination or by SARS-CoV-2 infection.

** Close contact is typically defined as being within 6 feet for at least 15 minutes (cumulative over a 24-hour period). However, close contact depends on the exposure level and setting (e.g., in the setting of an aerosol-generating procedure in a health care settings without proper personal protective equipment, close contact might be defined as any duration).

### Scope and Applicability 

The CSTE/CDC MIS-C surveillance case definition is intended for public health surveillance (e.g., case ascertainment and reporting). It establishes clinical, laboratory, and epidemiological reporting criteria for identification and classification of cases as confirmed, probable, or suspect MIS-C associated with SARS-CoV-2 infection. The case definition prioritizes features of MIS-C that distinguish it from similar pediatric inflammatory conditions and includes criteria that nonclinical surveillance staff at state, local, and territorial health departments can apply. This surveillance case definition might not capture the full range of presentations of MIS-C and was not developed as diagnostic criteria for MIS-C. Thus, it might not identify all MIS-C cases and is not intended to replace clinical judgment or inform patient management decisions (*19*). Although MIS-C is not a nationally notifiable condition and reporting is voluntary, health departments are encouraged to report all cases meeting confirmed, probable, or suspect criteria to CDC.

## Methods

### Revising the 2020 CDC MIS-C Case Definition

The process for revising the 2020 CDC MIS-C case definition and developing the CSTE/CDC MIS-C surveillance case definition included three approaches. These three approaches were 1) convening consultants with multidisciplinary expertise, 2) reviewing published literature and unpublished data on the MIS-C phenotype, and 3) evaluating different criteria through retrospective application to MIS-C national surveillance data.

#### Input from Consultants with Multidisciplinary Expertise 

To solicit individual expert input^†^ and feedback on proposed elements of the case definition, CDC convened two teleconferences in July and August 2021, comprising clinical experts in general pediatrics and pediatric rheumatology, infectious disease, cardiology, critical care, and immunology, as well as clinicians and surveillance experts from CSTE and WHO. Invited experts described relevant clinical and surveillance experiences and were asked to provide feedback on five topic areas regarding case definition criteria that were identified as contributing to the complexity of the case definition or that had been mentioned to CDC by health department reporting partners as considerations for inclusion in a future case definition. These five topic areas were 1) inclusion of nonhospitalized patients; 2) requirement of a minimum fever duration; 3) inclusion of clinical criteria related to thrombotic events, suppression of hematopoietic cell lines, and neurologic signs and symptoms; 4) selection of laboratory markers of systemic inflammation and appropriate thresholds for elevation; 5) and considerations for SARS-CoV-2 laboratory test criteria.

#### Published Literature and Unpublished Data on the MIS-C Phenotype

To better characterize features of MIS-C in comparison with similar conditions, the CSTE/CDC work group reviewed pertinent studies comparing the MIS-C phenotype with that of other pediatric hyperinflammatory conditions including COVID-19, Kawasaki disease, and toxic shock syndrome. Six studies published between May 14, 2020, and May 1, 2022, with large MIS-C cohorts or groups (at least 100 patients) were considered; four were U.S. cohort studies and two were meta-analyses that each included patients from nine countries (*16*,*17*,*20*–*23*). In addition, data from a comparison of two cohorts of MIS-C patients enrolled using the 2020 CDC (U.S. cohort) and WHO MIS-C case definitions (international cohort, 34 countries) were reviewed; these data were unpublished at the time of review but have since been published (*24*). Data from these sources were used to identify components of the 2020 CDC MIS-C case definition that were most effective in distinguishing MIS-C from other conditions and, conversely, those that might have contributed to misclassification.

Comparative U.S. cohort studies of MIS-C (subjects were enrolled using the 2020 CDC MIS-C case definition) and pediatric COVID-19 have demonstrated that mucocutaneous, cardiovascular, and hematologic organ system involvement as well as abdominal pain and, to a lesser extent, vomiting and diarrhea, are features that raise the likelihood of an MIS-C diagnosis (*17*,*20*). On the other hand, respiratory system involvement is much more common in children with a COVID-19 diagnosis; when respiratory system involvement occurs in MIS-C, it is often associated with cardiovascular involvement as well (*17*,*20*). One study, which included hospitalized children with MIS-C or COVID-19 diagnoses, identified three groups of phenotypically distinct SARS-CoV-2–associated illness (*16*). One group included two thirds of all patients with MIS-C diagnoses, particularly those who were previously healthy, who had gastrointestinal symptoms, manifested predominantly cardiovascular or mucocutaneous involvement or both, and had positive SARS-CoV-2 serology results in the absence of positive nucleic acid amplification test (NAAT) (e.g., polymerase chain reaction [PCR]) results. A second group included younger patients generally without underlying conditions who were less critically ill than those in the other two clusters and with positive NAAT results; this group included approximately 15% of all patients in whom MIS-C was diagnosed. The third group included primarily patients with underlying conditions and signs and symptoms associated with severe COVID-19, including abnormal chest radiographs, development of acute respiratory distress syndrome, and positive NAAT results. Although COVID-19 was diagnosed in most patients in this third group, the group also included nearly 20% of all patients in whom MIS-C was diagnosed. The authors concluded that, in addition to phenotypic heterogeneity among MIS-C cases, application of the 2020 CDC MIS-C case definition likely resulted in some misclassification of patients with severe COVID-19 as having MIS-C, particularly through its inclusion of respiratory system involvement. Despite their inclusion in enrollment criteria using the 2020 CDC MIS-C case definition, neurologic and renal involvement appeared to be equally common in children with MIS-C and COVID-19 (*20*,*21*).

Two observational cohort studies evaluated clinical outcomes in patients with MIS-C who were treated with intravenous immunoglobulin and corticosteroids compared with intravenous immunoglobulin alone (*25**,**26*). The first study (U.S. Overcoming COVID-19 Network MIS-C registry) enrolled patients meeting the 2020 CDC MIS-C case definition, and the second international study (Best Available Treatment Study) used the WHO MIS-C case definition that does not include respiratory, neurologic, or renal involvement and requires ≥3 days of fever (*13*). Each study had different conclusions regarding the benefit of concomitant initiation with intravenous immunoglobulin and corticosteroids (*25**,**26*). To evaluate whether the use of different MIS-C case definitions could have contributed to these contrasting findings, a third study was done to compare the cohort phenotypes (*24*). Despite differences in organ system criteria between the MIS-C definitions used for enrollment, approximately 90% of patients in both cohorts met both definitions. Moreover, among patients in the U.S. Overcoming COVID-19 Network MIS-C registry cohort, the most common reason for not meeting the WHO definition did not pertain to organ system involvement but rather to fever duration of <3 days (5% of patients). Overall organ system involvement was similar between cohorts, supporting a common set of MIS-C manifestations; no significant difference was found in the proportion of patients with CDC-defined respiratory, neurologic, or renal involvement, which were the least commonly involved organ systems in both cohorts.

In addition to pediatric COVID-19, cases of Kawasaki disease and toxic shock syndrome, both associated with organ dysfunction and systemic inflammation, might be misclassified as MIS-C. These conditions often are considered as possible diagnoses for patients under investigation for MIS-C. MIS-C has been distinguished from Kawasaki disease by having a higher prevalence of cardiovascular involvement, thrombocytopenia, abdominal pain, and headache, whereas MIS-C has been distinguished from toxic shock syndrome by more frequent conjunctival injection and less frequent rash, acute respiratory distress syndrome, and shock (*17*).

Laboratory tests vary in ability to distinguish MIS-C from pediatric COVID-19, Kawasaki disease, and toxic shock syndrome. Among laboratory markers of systemic inflammation, elevation in C-reactive protein (CRP) has been consistently greater in MIS-C patients than in children with COVID-19 and Kawasaki disease (*16*,*17*,*20*,*22*,*23*). To a lesser extent, CRP elevation also might be greater in MIS-C than in toxic shock syndrome (*17*). Most patients with MIS-C have CRP elevation of >3.0 mg/dL, the cutoff used in the American Heart Association Kawasaki Disease guideline to indicate ongoing systemic inflammation in patients suspected to have Kawasaki disease (*27*). Evidence is mixed regarding the utility of other inflammatory markers (e.g., ferritin, erythrocyte sedimentation rate, and procalcitonin) in distinguishing MIS-C from COVID-19, Kawasaki disease, and toxic shock syndrome (*16*,*17*,*22*,*23*). Other laboratory test results that were more elevated in MIS-C than in COVID-19 included absolute neutrophil count, markers of coagulopathy D-dimer and fibrinogen, and cardiac biomarkers troponin, brain natriuretic peptide (BNP), and N-terminal pro-BNP (NT-proBNP) (*16**,**17**,**20**,**22**,**23*). Absolute lymphocyte count and platelet count have been observed to be lower in MIS-C than in Kawasaki disease (*16*,*17*,*20*,*22*,*23*).

#### National MIS-C Surveillance Data and Analysis 

To estimate the effect of individual and combined changes to the 2020 CDC MIS-C case definition on case ascertainment, the work group performed a retrospective analysis of national MIS-C surveillance data. A data set was created from cases reported to CDC by state, local, and territorial health departments and adjudicated by CDC surveillance staff as meeting the 2020 CDC MIS-C case definition using variables from the standardized case report form. All cases meeting the 2020 CDC MIS-C case definition with MIS-C illness onset date on or before June 17, 2022, that were reported on or before August 31, 2022, were included.

The frequency of demographic variables, clinical features, laboratory findings, treatments administered, and short-term outcomes among all patients were calculated in the analytic set. The proportion with fever duration of ≥2 days and ≥3 days, the proportion with CRP elevation, and the proportion meeting various combinations of organ system involvement criteria, including those defined in the 2020 CDC MIS-C case definition, were calculated.

Each proportion of previously reported MIS-C cases captured through positive NAAT or antigen tests, positive SARS-CoV-2 serology, or epidemiologic link to a COVID-19 case were characterized. The analytic set was stratified into the following four mutually exclusive groups: 1) NAAT or antigen result positive and serology positive, 2) NAAT or antigen result positive and serology negative or not recorded, 3) both NAAT and antigen results negative or not recorded and serology positive, and 4) both NAAT and antigen results negative or not recorded and serology negative or not recorded (i.e., epidemiologic link only). Case counts in each group were compared.

All MIS-C criteria included in what would become the approved CSTE/CDC MIS-C surveillance case definition were then applied to the analytic set. The national MIS-C surveillance system did not record data from death certificates; therefore, vital records criteria corresponding to suspect case classification were not considered. Because dates often were missing for SARS-CoV-2 laboratory testing, timing constraints were not imposed on NAAT, antigen, or serology test results relative to MIS-C illness onset. The proportion of the analytic set that met the CSTE/CDC MIS-C surveillance case definition was calculated, excluding cases without a reported quantitative CRP measurement. The proportion of cases meeting criteria also was calculated after each of the following adjustments: requiring ≥2 days of fever (temperature ≥38°C or report of subjective fever), inclusion of an elevated ferritin result as an indicator of systemic inflammation in addition to CRP of ≥3.0 mg/dL, and re-addition of certain individual organ system criteria (respiratory, neurologic, and renal criteria [as defined in the 2020 CDC MIS-C case definition] and BNP or NT-proBNP elevation).

Finally, patient demographics and clinical and laboratory characteristics were compared between cases that did and did not meet the CSTE/CDC MIS-C surveillance case definition, excluding cases without a reported quantitative CRP measurement. The chi-square test was used to compare categorical variables, and the Wilcoxon rank-sum test was used to compare ordinal or continuous variables. P-values <0.05 were considered statistically significant for all statistical tests. All analyses were performed using SAS (SAS Institute; version 9.4).

##### National MIS-C Surveillance Findings 

Of 9,190 patients reported as possibly having MIS-C on or before August 31, 2022, a total of 325 were excluded that did not meet the 2020 CDC MIS-C case definition and 39 were excluded with illness onset date missing or after June 17, 2022. The remaining 8,826 adjudicated MIS-C cases in the analytic set were reported from 50 state health departments, the District of Columbia, Guam, New York City, Puerto Rico, and the U.S. Virgin Islands.

The 2020 CDC MIS-C case definition requires fever duration of ≥24 hours; 95.8% of cases were reported as having ≥2 days of fever and 89.5% had ≥3 days (Table 1). The most common organ system involvement per the 2020 CDC MIS-C case definition was hematologic, with only one case not meeting this criterion. By comparison, gastrointestinal involvement was reported in 92.7% of cases, cardiac in 89.3%, dermatologic in 72.5%, respiratory in 69.3%, neurologic in 49.6%, and renal in 18.8%.

**TABLE 1:** Numbers and percentages of cases of multisystem inflammatory syndrome in children,* by demographic and clinical characteristics

Characteristic	MIS-C cases (n = 8,826) No. (%)
**Age group (yrs)^†^**	
<1	285 (3.2)
1–4	1,884 (21.3)
5–11	4,107 (46.5)
12–15	1,680 (19.0)
16–20	869 (9.8)
**Age (yrs), median (IQR)^†^**	9 (5–13)
**Sex^§^**	
Female	3,489 (39.5)
Male	5,334 (60.5)
**Race and ethnicity** ^¶^	
Asian	219 (2.6)
Black	2,539 (30.5)
Hispanic or Latino	2,193 (26.3)
White	2,897 (34.8)
Other/multiple race	479 (5.8)
**Underlying medical condition**	
Obesity**	2,116 (26.1)
Chronic lung disease including asthma	574 (6.5)
**Clinical feature**	
Fever duration ≥2 days^††^	8,107 (95.8)
Fever duration ≥3 days^††^	7,569 (89.5)
**Organ system involvement per 2020 CDC MIS-C case definition**	
Hematologic	8,825 (100.0)
Gastrointestinal	8,181 (92.7)
Cardiac	7,878 (89.3)
Dermatologic	6,402 (72.5)
Respiratory	6,113 (69.3)
Neurologic	4,382 (49.6)
Renal	1,661 (18.8)
**Treatment**	
Intravenous immunoglobulins	7,355 (83.3)
Systemic corticosteroids	7,068 (80.1)
Other immunomodulatory treatment^§§^	1,895 (21.5)
No immunomodulatory treatment reported	415 (4.7)
Extracorporeal membrane oxygenation	120 (1.4)
**Outcome**	
Total days in hospital, median (IQR)^¶¶^	5 (4–8)
Intensive care admission	5,091 (57.7)
Total days in intensive care unit, median (IQR)***	3 (2–5)
Death	71 (0.8)

**Abbreviations:** IQR = interquartile range; MIS-C = multisystem inflammatory syndrome in children.

* Cases among persons aged<21 years reported to CDC on or before August 31, 2022, with illness onset date on or before June 17, 2022.

^† ^Excludes one case with missing age.

**^§^** Excludes three cases with missing sex.

^¶^ Percentages were calculated among 8,327 persons with known race and ethnicity. Racial and ethnic classifications followed CDC’s Office of Minority Health and Health Equity guidance. Non-Hispanic ethnicity was assumed if Hispanic or Latino ethnicity was not noted. Hispanic or Latino ethnicity was top-coded over Asian, Black, White, other race, and multiple races. American Indian or Alaska Native and Native Hawaiian or other Pacific Islander populations were represented as such, regardless of ethnicity. Because of small numbers, the category labeled Other/multiple race includes American Indian or Alaska Native, Native Hawaiian or other Pacific Islander, non-Hispanic other race, and non-Hispanic multiple races.

** Excludes 722 cases among children aged ≤2 years. Obesity was defined by recorded diagnosis or by body mass index using national reference standards (https://www.cdc.gov/nccdphp/dnpao/growthcharts/resources/sas.htm).

^††^ Excludes 365 cases with missing fever duration.

^§§^ Anakinra, bamlanivimab, canakinumab, COVID-19 convalescent plasma, cultured human marrow–derived mesenchymal stem cells (remestemcel-L), eculizumab, etanercept, hydroxychloroquine, infliximab, mycophenolate mofetil, pentoxifylline, ruxolitinib, tacrolimus, or tocilizumab.

^¶¶^ Excludes 404 cases with missing length of hospital stay.

*** Includes 3,530 cases with reported length of stay in intensive care unit.

Among reported MIS-C cases, 50.7% had a positive SARS-CoV-2 antibody test result without a positive NAAT or antigen test result (Table 2). By comparison, only 12.7% had a positive NAAT or antigen test result in the absence of a positive antibody test and 34.7% had positive results for both. The remaining 1.9% met the 2020 MIS-C case definition through epidemiologic link with a COVID-19 case in the absence of positive SARS-CoV-2 laboratory testing.

**TABLE 2:** Evaluation of SARS-CoV-2 laboratory testing in cases of multisystem inflammatory syndrome in children*

All testing	NAAT positive or antigen positive No. (%)	NAAT negative or not reported^†^ and antigen negative or not reported^†^ No. (%)	Total No. (%)
Antibody positive	3,061 (34.7)	4,477 (50.7)	**7,538 (85.4)**
Antibody negative or not reported^†^	1,123 (12.7)	165^§^ (1.9)	**1,288 (14.6)**
**Total**	**4,184 (47.4)**	**4,642 (52.6)**	**8,826 (100.0)**

**Abbreviations:** MIS-C = multisystem inflammatory syndrome in children; NAAT = nucleic acid amplification test.

* Cases among persons aged <21 years were reported to CDC on or before August 31, 2022, with illness onset date on or before June 17, 2022. Cases were to have a positive NAAT, antigen, or serology test, or exposure to a suspected or confirmed COVID-19 case within the 4 weeks before symptom onset (epidemiologic link).

^†^ Because of limitations in national MIS-C case reporting, negative laboratory results could not be distinguished from absence of a test performed or reported.

^§^ Cases with an epidemiologic link to a case of COVID-19, but no positive SARS-CoV-2 testing.

Overall, 8,516 (96.5%) MIS-C cases were reported with a CRP result, including those marked as normal or elevated in the absence of a quantitative value; 7,081 (80.2%) were reported with a quantitative result. Of those with a quantitative CRP result, 93.7% had CRP of ≥3.0 mg/dL, 91.7% had involvement of two or more organ systems per the CSTE/CDC MIS-C surveillance case definition, and 6,158 (87.0%) met the CSTE/CDC MIS-C surveillance case definition, including 6,051 (85.5%) that met confirmed case criteria and 107 (1.5%) that met probable case criteria (Table 3). When required fever duration of ≥2 days was added to the CSTE/CDC MIS-C surveillance case definition, the percentage of reported MIS-C cases that met criteria decreased to 82.3%. Conversely, including ferritin as an additional inflammatory marker with CRP increased this percentage to 92.4%. Individually adding respiratory, neurologic, and renal criteria as defined in the 2020 CDC MIS-C case definition increased case capture to 90.1%, 89.1%, and 87.3%, respectively. Including elevation in BNP or NT-proBNP in cardiac criteria increased case capture to 89.8%.

**TABLE 3:** Numbers and percentages of cases of multisystem inflammatory syndrome in children with a quantitative C-reactive protein result*

Criteria	MIS-C cases with a quantitative CRP result (n = 7,081)^†^ No. (%)
**CSTE/CDC MIS-C surveillance case definition component**	
CRP ≥3.0 mg/dL	6,635 (93.7)
**Organ system involvement per CSTE/CDC MIS-C surveillance case definition**	
At least two organ systems involved	6,492 (91.7)
Gastrointestinal	6,316 (89.2)
Mucocutaneous	5,260 (74.3)
Cardiac	4,381 (61.9)
Hematologic	3,964 (56.0)
Shock	3,003 (42.4)
Met CSTE/CDC MIS-C surveillance case definition (confirmed or probable)^§^	6,158 (87.0)
Met confirmed case criteria^§^	6,051 (85.5)
Met probable case criteria^§^	107 (1.5)
More inclusive adjustment	
+ Elevated ferritin as an additional inflammatory marker with CRP ≥3.0 mg/dL	6,543 (92.4)
+ Respiratory involvement^¶^ included in multisystem criteria	6,378 (90.1)
+ Elevated BNP or NT-proBNP included in multisystem criteria, within cardiac	6,357 (89.8)
+ Neurologic involvement^¶^ included in multisystem criteria	6,306 (89.1)
+ Renal involvement^¶^ included in multisystem criteria	6,180 (87.3)
More exclusive adjustment	
+ Requirement of ≥2 days of fever	5,829 (82.3)

**Abbreviations:** BNP = brain natriuretic peptide; CRP = C-reactive protein; CSTE = Council of State and Territorial Epidemiologists; MIS-C = multisystem inflammatory syndrome in children; NT-proBNP = N-terminal pro-BNP.

* Cases among persons aged <21 years reported to CDC on or before August 31, 2022, with illness onset date on or before June 17, 2022, meeting the CSTE/CDC MIS-C surveillance case definition single components and meeting alternative combined MIS-C criteria incorporating individual adjustments to make the definition either more inclusive or exclusive of cases.

^†^ Excludes 1,745 of 8,826 total cases that did not have a recorded quantitative CRP result.

^§^ Positive SARS-CoV-2 nucleic acid amplification test, antigen, and serology results were accepted regardless of timing relative to hospitalization.

^¶^ Defined according to 2020 CDC MIS-C case definition.

Among MIS-C cases with a quantitative CRP result meeting the CSTE/CDC MIS-C surveillance case definition, patient age was more often in middle childhood and early adolescence (patients aged 5–15 years) and less often in early childhood or later adolescence (patients aged <5 years or 16–20 years), compared with cases that did not meet the case definition (Table 4). Although the CSTE/CDC MIS-C surveillance case definition includes no required fever duration, cases meeting this definition were more likely than those not meeting it to have fever duration of ≥2 days and ≥3 days. When the addition of a ≥2-day fever requirement was assessed as a potential case definition criterion, patients with coronary artery abnormalities, patients admitted to intensive care, and patients who received extracorporeal membrane oxygenation treatment were overrepresented among excluded cases (data not shown). The work group was unable to assess whether this might have been because of limited information available to surveillance staff regarding number of days with fever before hospitalization or whether these severely ill patients might have been recognized and treated early with immunomodulatory agents, thus reducing their recorded fever duration.

**TABLE 4:** Characteristics of cases meeting or not meeting the CSTE/CDC MIS-C surveillance case definition* — National MIS-C Surveillance, United States

Characteristic	Previously reported MIS-C cases meeting CSTE/CDC MIS-C standardized surveillance case definition^†^ (n = 6,158) No. (%)	Previously reported MIS-C cases not meeting CSTE/CDC MIS-C standardized surveillance case definition^†,§^ (n = 923) No. (%)	p-value^¶^
**Age group (yrs)**			<0.001
<1	141 (2.3)	95 (10.3)	
1–4	1,208 (19.6)	285 (30.9)	
5–11	3,020 (49.0)	275 (29.8)	
12–15	1,216 (19.7)	136 (14.7)	
16–20	573 (9.3)	132 (14.3)	
Sex**			0.001
Female	2,382 (38.7)	410 (44.5)	
Male	3,775 (61.3)	512 (55.5)	
**Race and ethnicity** ^††^			0.005^§§^
Asian	148 (2.5)	27 (3.1)	
Black	1,773 (30.4)	226 (26.3)	
Hispanic or Latino	1,510 (25.9)	264 (30.7)	
White	2,072 (35.5)	289 (33.6)	
Other/multiple race	335 (5.7)	53 (6.2)	
**Underlying medical condition**			
Obesity^¶¶^	1,545 (26.7)	214 (29.8)	0.08
Chronic lung disease including asthma	421 (6.9)	59 (6.4)	0.62
**Clinical feature**			
Fever duration ≥2 days***	5,829 (96.4)	843 (94.4)	0.003
Fever duration ≥3 days***	5,494 (90.9)	748 (83.8)	<0.001
CRP ≥3.0 mg/dL	6,158 (100.0)	477 (51.7)	—^†††^
Positive SARS-CoV-2 NAAT or antigen result	2,872 (46.6)	522 (56.6)	<0.001
Positive SARS-CoV-2 antibody test result	5,442 (88.4)	635 (68.8)	<0.001
**Organ system involvement per 2020 CDC MIS-C case definition**			
Hematologic	6,158 (100.0)	923 (100.0)	—
Gastrointestinal	5,942 (96.5)	731 (79.2)	<0.001
Cardiac	5,797 (94.1)	662 (71.7)	<0.001
Dermatologic	4,865 (79.0)	395 (42.8)	<0.001
Respiratory	4,412 (71.6)	599 (64.9)	<0.001
Neurologic	3,322 (53.9)	336 (36.4)	<0.001
Renal	1,295 (21.0)	81 (8.8)	<0.001
**Multisystem involvement per CSTE/CDC MIS-C surveillance case definition**			
Gastrointestinal	5,715 (92.8)	601 (65.1)	—
Mucocutaneous	4,865 (79.0)	395 (42.8)	—
Cardiac	4,196 (68.1)	185 (20.0)	—
Troponin elevation	3,599 (58.4)	152 (16.5)	—
Left ventricular ejection fraction <55%	1,653 (26.8)	46 (5.0)	—
Coronary artery dilatation, aneurysm, or ectasia	912 (14.8)	44 (4.8)	—
Hematologic	3,760 (61.1)	204 (22.1)	—
Shock	2,884 (46.8)	119 (12.9)	—
**Treatment**			
Intravenous immunoglobulins	5,468 (88.8)	546 (59.2)	<0.001
Systemic corticosteroids	5,156 (85.4)	610 (69.4)	<0.001
Other immunomodulatory treatment^§§§^	1,511 (25.7)	134 (15.8)	<0.001
No immunomodulatory treatment reported	180 (2.9)	153 (16.6)	<0.001
Extracorporeal membrane oxygenation	87 (1.4)	6 (0.7)	0.06
**Outcome**			
Total days in hospital, median (IQR)^¶¶¶^	5 (4–8)	4 (3–6)	<0.001
Intensive care admission	3,802 (61.7)	334 (36.2)	<0.001
Total days in intensive care unit, median (IQR)****	3 (2–5)	3 (2–6)	0.27
Death	47 (0.8)	7 (0.8)	0.99

**Abbreviations:** CRP = C-reactive protein; CSTE = Council of State and Territorial Epidemiologists; IQR = interquartile range; MIS-C = multisystem inflammatory syndrome in children; NAAT = nucleic acid amplification test.

* Cases among persons aged <21 years reported to CDC on or before August 31, 2022, with illness onset date on or before June 17, 2022.

^†^ Positive SARS-CoV-2 NAAT, antigen, and serology results were accepted regardless of timing relative to hospitalization.

^§^ Excludes 1,745 cases without a recorded quantitative CRP result.

^¶^ The chi-square test was used to compare categorical variables, and the Wilcoxon rank-sum test was used to compare ordinal or continuous variables.

** Excludes two cases with missing sex (one in each column).

^††^ Percentages were calculated among 6,697 persons with known race and ethnicity. Racial and ethnic classifications followed CDC’s Office of Minority Health and Health Equity guidance. Non-Hispanic ethnicity was assumed if Hispanic or Latino ethnicity was not noted. Hispanic or Latino ethnicity was top-coded over Asian, Black, White, other race, and multiple races. American Indian or Alaska Native and Native Hawaiian or other Pacific Islander populations were represented as such, regardless of ethnicity. Because of small numbers, the category labeled Other/multiple race includes American Indian or Alaska Native, Native Hawaiian or other Pacific Islander, non-Hispanic other race, and non-Hispanic multiple races.

^§§^ Chi-square test, excluding Other/multiple race.

^¶¶^ Excludes 586 cases among children aged ≤2 years. Obesity was defined by recorded diagnosis or by body mass index using national reference standards (https://www.cdc.gov/nccdphp/dnpao/growthcharts/resources/sas.htm).

*** Excludes 144 cases with missing fever duration.

^†††^ Statistical comparison was not performed for criteria included in the CSTE/CDC MIS-C surveillance case definition, but not included in the 2020 CDC MIS-C case definition.

^§§§^ Anakinra, bamlanivimab, canakinumab, COVID-19 convalescent plasma, cultured human marrow-derived mesenchymal stem cells (remestemcel-L), eculizumab, etanercept, hydroxychloroquine, infliximab, mycophenolate mofetil, pentoxifylline, ruxolitinib, tacrolimus, or tocilizumab.

^¶¶¶^ Excludes 212 cases with missing length of hospital stay.

**** Includes 2,940 cases with reported length of stay in intensive care unit.

Patients whose illness met the CSTE/CDC MIS-C surveillance case definition were more likely to have a positive SARS-CoV-2 serology test, and less likely to have a positive NAAT or antigen test, than those who did not meet the definition. These patients also were more likely to have received intravenous immunoglobulin and systemic corticosteroids and were less likely to have received no immunomodulatory therapy. They were more likely to require intensive care admission and had longer length of hospitalization; however, mortality was similar to that of patients with illnesses not meeting the CSTE/CDC MIS-C surveillance case definition. Organ system criteria from both the 2020 CDC MIS-C case definition and the CSTE/CDC MIS-C surveillance case definition were more often met among cases that met the CSTE/CDC MIS-C surveillance case definition. The only exception was hematologic involvement as defined in the 2020 CDC MIS-C case definition, which was present in all evaluated cases.

#### Additional Considerations 

The CSTE/CDC MIS-C surveillance case definition was based not only on data-driven approaches and expert opinion, but also on practical case reporting considerations and anticipated future surveillance challenges. For instance, a ≥2-day fever requirement only slightly reduced retrospective case ascertainment compared with accepting any fever and would likely increase definition specificity. However, requiring surveillance partners to confirm fever duration would entail more detailed review of medical records, because exact dates of fever onset and duration are difficult to ascertain and might not be well documented. This would place increased workload on reporting partners and might reduce participation in the national passive surveillance system. In addition, as MIS-C recognition increases and therapeutics improve, more patients might receive early immunomodulatory treatment, thereby shortening fever duration. For these reasons, the work group determined that accepting fever of any duration was preferable to imposing a minimum duration in the case definition.

Similarly, although including BNP or NT-proBNP elevation slightly increased retrospective case ascertainment and might increase sensitivity, confirming elevation in these laboratory tests is challenging for nonmedically trained surveillance staff because of varying reference ranges used by hospital and clinical laboratories. Compounding the difficulty in assigning a threshold for elevation, these biomarkers might become moderately elevated in response to aggressive intravenous fluid resuscitation in children with hypotension or shock (*28*).

The work group considered expanding the case definition to include nonhospitalized patients, who might have milder illness compatible with a spectrum of MIS-C. However, for MIS-C, the objective was to update a surveillance case definition that would ascertain severe clinical events of public health significance, those that would require the most resources of the health care system, and those that would be feasible and practical for reporting. Expanding the case definition to include nonhospitalized patients would vastly increase the scope of MIS-C surveillance, would likely require different case ascertainment procedures by state, local, territorial, and tribal health departments, and might reduce participation in the surveillance system. For these reasons, hospitalization was retained as a requirement in the CSTE/CDC MIS-C surveillance case definition.

Consideration also was given to eliminating the provision for epidemiologic link to a COVID-19 case and simply requiring laboratory evidence of SARS-CoV-2 infection. However, although cases without SARS-CoV-2 laboratory evidence have made up a small proportion of reported MIS-C cases to date, this might change in the future. Widespread seropositivity among U.S. children might result from increased COVID-19 vaccination coverage and previous SARS-CoV-2 infection, decreasing the clinical utility of serology testing; a positive result might not indicate that the patient’s illness is related to SARS-CoV-2. If clinicians perform serology testing less often, many children with MIS-C might not meet a stringent requirement of a positive SARS-CoV-2 laboratory test. In addition, although the work group considered a requirement of a positive nucleocapsid-specific antibody test result for vaccinated patients, this was not included in the CSTE/CDC MIS-C surveillance case definition because the antigen specificity (i.e., anti-nucleocapsid versus anti-spike) of antibody tests might not always be available to health department staff. Finally, on the basis of data indicating that >95% of persons with a history of COVID-19 had illness onset within 60 days before MIS-C onset, a 60-day window before hospitalization for MIS-C was implemented during which positive SARS-CoV-2 NAAT or antigen results could count toward meeting the CSTE/CDC MIS-C surveillance case definition (*9*).

Vital records criteria were included in the CSTE/CDC MIS-C surveillance case definition, corresponding to suspect case classification, to facilitate reporting of out-of-hospital deaths of children with a compatible illness and exposure history. Many of the clinical criteria are challenging to ascertain without advanced diagnostic capabilities available only in inpatient settings (e.g., urgent echocardiography), and therefore cannot be confirmed in persons who die outside of the hospital. Reporting such deaths that could be attributable to MIS-C is important to public health surveillance and is facilitated through inclusion of vital records criteria.

## Public Health Implications

Ongoing national surveillance for MIS-C associated with SARS-CoV-2 infection requires a standardized surveillance case definition that incorporates knowledge about the cardinal features of MIS-C and better distinguishes it from other hyperinflammatory syndromes in children. This CSTE/CDC MIS-C surveillance case definition, for cases with MIS-C illness onset on or after January 1, 2023, fills that need and will be used to estimate disease burden, monitor geographic trends, and characterize the demographic characteristics of persons affected by MIS-C associated with SARS-CoV-2 infection in the United States. Evaluation of trends before and after January 1, 2023, will need to consider the change in surveillance case definition. Continued surveillance will be crucial as new SARS-CoV-2 variants of concern emerge and circulate in the United States and vaccination recommendations expand to include younger children, potentially altering the epidemiology of MIS-C.

As SARS-CoV-2 seroprevalence increases in the pediatric population because of previous infection and COVID-19 vaccination, application of the CSTE/CDC MIS-C surveillance case definition will need to be interpreted in the context of possible alternative diagnoses. Children with bacterial sepsis, tickborne illness, and rheumatologic conditions, including Kawasaki disease, might meet most components of the definition and incidentally have a positive serologic result. Surveillance procedures to record cases of MIS-C should involve review of medical records whenever possible, with particular attention to the ultimate diagnosis by the clinical treatment team.

The CSTE/CDC MIS-C surveillance case definition does not include all manifestations of MIS-C that distinguish it from other conditions; certain clinical features are impractical for surveillance programs to review. For this reason, the case definition is not designed as a set of diagnostic criteria, and its direct application in clinical care might miss cases of MIS-C. Clinicians should use all available clinical features, laboratory results, and imaging studies in diagnosis of MIS-C and for management decisions.

## Future Directions

CDC will continue to work with state, local, territorial, and tribal health departments to gather data and monitor trends in MIS-C case counts, characteristics, and patient outcomes over time. Changes to MIS-C epidemiology, clinician testing practices, or predominant SARS-CoV-2 variant might necessitate future updates to the surveillance case definition. Periodic analyses of national surveillance data will be performed to evaluate the need for an update.

The CSTE/CDC MIS-C surveillance case definition also will be implemented in active, hospital-based surveillance supported by CDC. As a complement to national passive surveillance, this activity will allow for detailed data collection about children hospitalized with MIS-C. Active surveillance will provide a unique opportunity to compare clinical features of children admitted to the participating surveillance network hospitals with hyperinflammatory conditions that meet either or both the 2020 MIS-C case definition and the CSTE/CDC MIS-C surveillance case definition.

## Footnotes

* The CSTE/CDC work group consisted of the author, coauthors, and CDC subject matter experts listed in the CSTE/CDC position statement (https://cdn.ymaws.com/www.cste.org/resource/resmgr/ps/ps2022/22-ID-02_MISC.pdf), and all are coauthors of this report.

† Procedures for convening experts (i.e., consultants) for the teleconferences were reviewed by the CDC Federal Advisory Committee Act (FACA) Program and were determined to meet the standard for FACA exemption for groups assembled to provide individual advice.
